# A new classification of the long-horned caddisflies (Trichoptera: Leptoceridae) based on molecular data

**DOI:** 10.1186/1471-2148-11-10

**Published:** 2011-01-12

**Authors:** Tobias Malm, Kjell Arne Johanson

**Affiliations:** 1 Entomology Department, Swedish Museum of Natural History, Box 50007, SE-104 05 Stockholm, Sweden; 2 Department of Zoology, Stockholm University, SE-106 09 Stockholm, Sweden

## Abstract

**Background:**

Leptoceridae are among the three largest families of Trichoptera (caddisflies). The current classification is founded on a phylogenetic work from the 1980's, based on morphological characters from adult males, *i.e*. wing venation, tibial spur formula and genital morphology. In order to get a new opinion about the relationships within the family, we undertook a molecular study of the family based on sequences from five genes, mitochondrial COI and the four nuclear genes CAD, EF-1α, IDH and POL.

**Results:**

The resulting phylogenetic hypotheses are more or less congruent with the morphologically based classification, with most genera and tribes recovered as monophyletic, but with some major differences. For monophyly of the two subfamilies Triplectidinae and Leptocerinae, one tribe of each was removed and elevated to subfamily status; however monophyly of some genera and tribes is in question. All clades except Leptocerinae, were stable across different analysis methods.

**Conclusions:**

We elevate the tribes Grumichellini and Leptorussini to subfamily status, Grumichellinae and Leptorussinae, respectively. We also propose the synonymies of *Ptochoecetis *with *Oecetis *and *Condocerus *with *Hudsonema*.

## Background

Being one of the three largest Trichoptera families, the long-horned caddisflies, Leptoceridae Leach, have received great interest since they were first described. In 1815, Leach [1] described the group as a tribe, Leptocerides, into which he included several *Phryganea *Linnaeus species from *Systema Naturae *[2]. Before the twentieth century the family included the now recognised individual families Beraeidae Wallengren, Calamoceratidae Ulmer, Molannidae Wallengren and Odontoceridae Wallengren [3]. Taxonomic work on this family has until now resulted in about 1,800 described species classified into 46 extant genera within two recognized subfamilies, Leptocerinae Leach and Triplectidinae Ulmer. The latest generic addition, *Osflintia *Calor & Holzenthal, was recently described [4]. Representatives of Leptocerinae can be found worldwide, while species of Triplectidinae occur mainly in the Australasian and Neotropical regions.

The first major systematic arrangements of the family were made in the early 20^th ^century. Ulmer [5] divided the genera into the two subfamilies, which he later revised [6] (Table 1A). Almost simultaneously, Silfvenius [7] had brought into use three tribes within Leptoceridae: Leptocerini Leach (incl. *Leptocerus *Leach), Mystacidini Burmeister (incl. *Erotesis *McLachlan, *Mystacides *Berthold and *Triaenodes *McLachlan) and Oecetini Silfvenius (incl. *Oecetis *McLachlan). In his revision of the Triplectidinae, Mosely [8] added 6 genera to the subfamily: *Atanatolica *Mosely, *Atriplectides *Mosely, *Hudsonema *Mosely, *Loticana *Mosely, *Notalina *Mosely and *Triplectidina *Mosely. *Loticana *was later synonymised with *Symphitoneuria *Ulmer and *Atriplectides *was placed in Odontoceridae [9], the latter genus was separated as a distinct family, Atriplectididae by Neboiss [10]. The tribe Athripsodini Morse & Wallace was erected for the genera *Athripsodes *Billberg and *Ceraclea *Stephens [11].

**Table 1:** Leptoceridae classifications during a century

A.					B.						C.				
**Ulmer, 1907**					**Morse, 1981**						**"Current classification"**				

Leptocerinae Leach, 1815					Leptocerinae Leach, 1815						Leptocerinae Leach, 1815				
	***Adicella ***McLachlan, 1877					Leptorussini Morse, 1981						Achoropsychini Holzenthal, 1984,			
	***Erotesis ***McLachlan, 1877						***Leptorussa ***Mosely, 1953						***Achoropsyche ***Holzenthal, 1984		
	***Homilia ***McLachlan, 1877					Athripsodini Morse & Wallace, 1976						Athripsodini Morse & Wallace, 1976			
	*Leptocella Banks, 1899						***Athripsodes ***Billberg, 1820						***Athripsodes ***Billberg, 1820		
		- (now synonym to *Nectopsyche*)					***Axiocerina ***Ross, 1957						***Axiocerina ***Ross, 1957		
	***Leptocerus ***Leach, 1815						***Ceraclea ***Stephens, 1829						***Ceraclea ***Stephens, 1829		
	***Mystacides ***Berthold, 1827						***Leptecho ***Barnard, 1934						***Homilia ***McLachlan, 1877		
	*Oecetinella Ulmer, 1907						***Leptocerina ***Mosely, 1932						***Leptecho ***Barnard, 1934		
		- (now synonym to *Oecetis*)					*Leptocerodes Lestage, 1936						***Leptoceriella ***Schmid, 1993		
	***Oecetis ***McLachlan, 1877							- (now synonym to *Athripsodes*)					***Leptocerina ***Mosely, 1932		
	*Oecetodes Ulmer, 1907					Nectopsychini Morse, 1981							***Neoathripsodes ***Holzenthal, 1989		
		- (now synonym to *Oecetis*)					***Nectopsyche ***Mueller, 1879					Blyzophilini Anderson, Kjaerandsen, & Morse, 1999			
	***Parasetodes ***McLachlan, 1880						***Parasetodes ***McLachlan, 1880						***Blyzophilus ***Anderson & Kjaerandsen, 1999		
	*Pseudoleptocerus Ulmer, 1907					Leptocerini Leach, 1815						Leptocerini Leach, 1815			
		- (now subgenus of *Ceraclea*)					***Leptocerus ***Leach, 1815						***Leptocerus ***Leach, 1815		
	*Pseudosetodes Ulmer, 1905					Triaenodini Morse, 1981						Leptorussini Morse, 1981			
		- (now synonym to *Oecetis*)					***Adicella ***McLachlan, 1877						***Leptorussa ***Mosely, 1953		
	***Setodes ***Rambur, 1842						*Allosetodes Banks, 1931					Mystacidini Burmeister, 1839			
	***Triaenodes ***McLachlan, 1865							- (now synonym to *Triaenodes*)					***Mystacides ***Berthold, 1827		
Triplectidinae Ulmer, 1906							***Erotesis ***McLachlan, 1877						***Fernandoschmidia ***Holzenthal & Andersen, 2007		
	*Notanatolica McLachlan, 1866						*Triaenodella Mosely, 1932						***Tagalopsyche ***Banks, 1913		
		- (now synonym to *Triplectides*)						- (now synonym to *Triaenodes*)				Nectopsychini Morse, 1981			
	***Symphitoneuria ***Ulmer, 1906						***Triaenodes ***McLachlan, 1865						***Nectopsyche ***Mueller, 1879		
	***Triplectides ***Kolenati, 1859						*Ylodes Milne, 1934						***Parasetodes ***McLachlan, 1880		
Appendix to the Leptocerinae								- (now synonym to *Triaenodes*)				Oecetini Silfvenius, 1905			
	***Grumichella ***Mueller, 1879					Oecetini Silfvenius, 1905							***Oecetis ***McLachlan, 1877		
							*Oecetinella Ulmer, 1907						***Ptochoecetis ***Ulmer, 1931		
								- (now synonym to *Oecetis*)				Setodini Morse, 1981			
							***Oecetis ***McLachlan, 1877						***Hemileptocerus ***Ulmer, 1922		
							*Oecetodella Ulmer, 1930						***Sericodes ***Schmid, 1987		
								- (now synonym to *Oecetis*)					***Setodes ***Rambur, 1842		
							*Paraoecetis Lestage, 1921						***Trichosetodes ***Ulmer, 1915		
								- (now synonym to *Oecetis*)				Triaenodini Morse, 1981			
							*Pseudosetodes Ulmer, 1905						***Adicella ***McLachlan, 1877		
								- (now synonym to *Oecetis*)					***Erotesis ***McLachlan, 1877		
							***Ptochoecetis ***Ulmer, 1931						***Triaenodes ***McLachlan, 1865		
							*Setodellina Lestage, 1919					*Incertae sedis *in Leptocerinae			
								- (now synonym to *Oecetis*)					***Amphoropsyche ***Holzenthal, 1985		
							*Setodina Banks, 1907						***Brachysetodes ***Schmid, 1955		
								- (now synonym to *Oecetis*)					***Poecilopsyche ***Schmid, 1968		
						Setodini Morse, 1981							***Russobex ***StClair, 1988		
							*Episetodes Martynov, 1936				Triplectidinae Ulmer, 1906				
								- (now synonym to *Trichosetodes*)				Grumichellini Morse, 1981			
							***Hemileptocerus ***Ulmer, 1922						***Amazonatolica ***Holzenthal & Oliveira Pes, 2004		
							***Setodes ***Rambur, 1842						***Atanatolica ***Mosely, 1936		
							***Trichosetodes ***Ulmer, 1915						***Gracilipsodes ***Sykora, 1967		
						Mystacidini Burmeister, 1839							***Grumichella ***Mueller, 1879		
							***Mystacides ***Berthold, 1827						***Osflintia ***Calor & Holzenthal, 2008		
							***Tagalopsyche ***Banks, 1913						***Triplexa ***Mosely, 1953		
						*Incertae sedis *in Leptocerinae						Hudsonemini Morse, 1981			
							***Brachysetodes ***Schmid, 1955						***Condocerus ***Neboiss, 1977		
							***Poecilopsyche ***Schmid, 1968						***Hudsonema ***Mosely, 1936		
					Triplectidinae Ulmer, 1906								***Notalina ***Mosely, 1936		
						Grumichellini Morse, 1981						Triplectidini Ulmer, 1906			
							***Atanatolica ***Mosely, 1936						***Lectrides ***Mosely, 1953		
							***Grumichella ***Mueller, 1879						***Notoperata ***Neboiss, 1977		
						Hudsonemini Morse, 1981							***Symphitoneuria ***Ulmer, 1906		
							***Condocerus ***Neboiss, 1977						***Symphitoneurina ***Schmid, 1950		
							***Hudsonema ***Mosely, 1936						***Triplectides ***Kolenati, 1859		
							***Notalina ***Mosely, 1936						***Triplectidina ***Mosely, 1936		
							***Triplexa ***Mosely, 1953						***Westriplectes ***Neboiss, 1977		
						Triplectidini Ulmer, 1906					*Incertae sedis *in Leptoceridae				
							***Lectrides ***Mosely, 1953						***Nietnerella ***Kimmins, 1963		
							***Notoperata ***Neboiss, 1977								
							***Symphitoneuria ***Ulmer, 1906								
							***Symphitoneurina ***Schmid, 1950								
							***Triplectides ***Kolenati, 1859								
							***Triplectidina ***Mosely, 1936								
							***Westriplectes ***Neboiss, 1977								

**Table 1A**. Classification of Leptoceridae by Ulmer [6]. Generic names in bold are considered valid and those with an asterisk are considered invalid (see parenthetical notation). **Table 1B**. Classification by Morse [12]. Bold names are considered valid and names in italics and with asterisk are considered invalid (see parenthetical notation). **Table 1C**. Classification modified from "World Trichoptera Check-list" http://entweb.clemson.edu/database/trichopt/hierarch.htm, illustrating the current understanding of the classification of extant genera (previous to this work).

**Figure 1:**
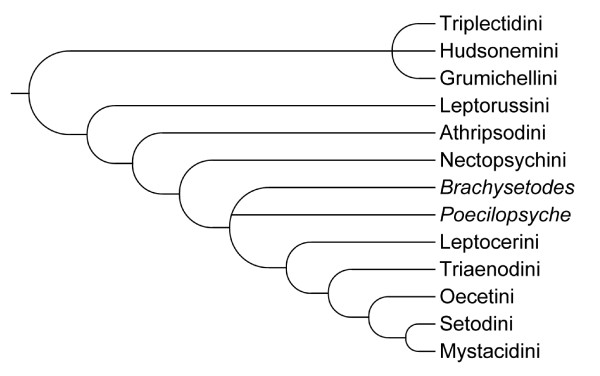
**Phylogeny of Leptoceridae tribes, after Morse, 1981**. Phylogeny based on morphological synapomorphies for each Leptoceridae tribe and two non-associated genera. After [12].

A natural classification of Leptoceridae was advocated by Morse [12] who arranged most of the known genera into tribal groups (Table 1B) based on proposed morphological synapomorphies, and also presented a hypothesis of the evolution within the family (Figure 1). In that phylogeny, the relationships were unresolved among the three tribes of Triplectidinae: Grumichellini Morse, Hudsonemini Morse and Triplectidini Ulmer. Leptocerinae were in turn divided into eight tribes (with the exception of a few genera): Leptorussini Morse, Athripsodini, Nectopsychini Morse, Leptocerini, Triaenodini Morse, Oecetini, Setodini Morse and Mystacidini. This phylogenetic hypothesis represents the backbone of the current Leptoceridae classification (Table 1C). Holzenthal [13] erected a new tribe within the Leptocerinae for the neotropical genus *Achoropsyche *Holzenthal. Holzenthal [13,14] stated that it represents the sister group to the Triaenodini-Mystacidini clade in Morse's (1981) phylogeny (Figure 1). The Triplectidinae were revised by Morse & Holzenthal [15], with the genera hierarchically ordered by synapomorphies in a tree; in contrast to Morse's work [12], the genus *Triplexa *Mosely was transferred to Grumichellini from Hudsonemini. The authors were not able to resolve the relationships among the Triplectidini genera *Lectrides *Mosely, *Symphitoneuria*, *Symphitoneurina *Schmid, *Triplectides *Kolenati and *Triplectidina*, and considered them as unresolved within *Triplectides s.l.*, questioning the monophyly of *Triplectides *without the inclusion of the other mentioned genera.

The latest addition of Leptoceridae tribes was the description of Blyzophilini Andersen, Kjærandsen & Morse for *Blyzophilus *Andersen & Kjaerandsen, a monotypic genus from Ghana [16]. Following the phylogeny of Morse [12], the tribe was placed in a trichotomy with Leptocerini and the remaining Leptocerinae. In his revision of the tribes Leptocerini and Setodini, Schmid [17] demonstrated that *Setodes *Rambur is paraphyletic, including the genera *Hemileptocerus *Ulmer, *Trichosetodes *Ulmer and *Sericodes *Schmid. The Indian genus *Nietnerella *Kimmins was described within Leptoceridae, but having morphological affinities to both Odontoceridae and Calamoceratidae. This uncertain position is presently maintained [3]. De Moor [18] described a larva from South Africa that is still unassociated with adults, but placed in Leptoceridae. It was regarded as a possible member of the Triplectidinae even though the morphological phylogeny did not place it in either of the subfamilies. A later analysis of the Leptocerinae, with more character data, included this larva but without explanation [19].

The Leptoceridae (represented by 12 species in 10 genera) were recovered as monophyletic in phylogenies presented by Kjer *et al. *[20,21] and Holzenthal *et al. *[22] based on a combination of molecular and morphological data. No phylogenetic patterns within the family were assumed or discussed.

### Characteristics of Leptoceridae

**Figure 2:**
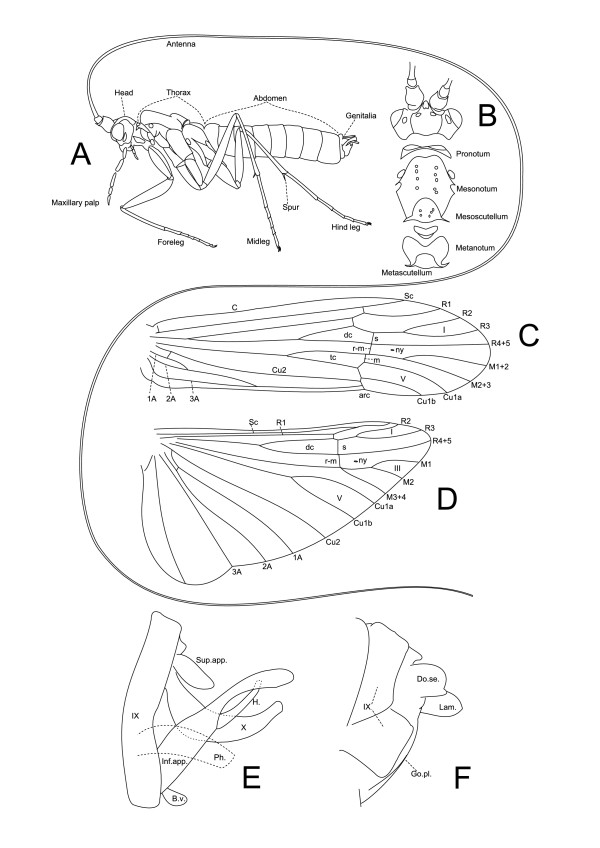
**Morphology of the Leptoceridae**. **Figure 2A**. *Gracilipsodes psocopterus *Sykora body, lateral view; modified from [31]. **Figure 2B**. *Gracilipsodes psocopterus *Sykora head + thorax, dorsal view; modified from [31]. **Figure 2C-D**. *Notoperata maculata *(Mosely) forewing (**C**) and hind wing (**D**), modified from [52]. Abbreviations: C - costal vein, Sc - subcostal vein, R1-5 - radial veins 1-5, M1-4 - median veins 1-4, Cu1-2 - cubital veins 1-2, 1-3A - anal veins 1-3, I - apical fork 1, III - apical fork 3, V - apical fork 5, dc - discoidal cell, tc - thyridial cell, ny - nygma, s - sectoral crossvein connecting R2 + 3 with R4+5, r-m - crossvein connecting R and M, m - crossvein connecting M and Cu. **Figure 2E-F**. *Notoperata maculata *(Mosely) male (**E**) and female (**F**) genitalia, modified from [52]. Abbreviations: IX - segment 9, X - tergum 10, Sup.app. - superior appendage, Inf.app. - inferior appendage, H - harpago, B.v. - basoventral lobe of inferior appendage, Do.se. - dorsal setose lobe, Lam. - lamella, Go.pl. - gonopod plate.

Adult members of the family (Figure 2) are recognised by having unusually long antennae, hence the common-name "long-horned caddisflies". The absences of ocelli and pre-apical mid-tibial spurs in combination with the presence of two bands of setiferous punctures along the dorsal side of the mesoscutum, instead of setal warts, are also characteristics of the family. The forewings are relatively narrow, while the hind wings may be narrow or broad, and in many genera within Leptocerinae and Grumichellini the forewings are variously spotted [3,6,23].

Larvae are recognised by their long antennae, long hind legs and the presence of at least two setae on the metasternum. The larval cases are built from a variety of materials, *e.g*. sand grains, plant fragments, twigs or silk. They are usually tubular, but flattened cases are not uncommon, and some cases are spiral (*Leptecho helicotheca *Scott). The larvae are mainly detritivorous shredders and periphyton scrapers, and some are predators. The larvae of certain *Ceraclea *species are reported to feed on freshwater sponges [24-27].

The pupae are slender, with long antennae wound up around abdomen, and without distal modification of the mandibles [28].

#### Subfamily Triplectidinae

Three synapomorphies for this subfamily were suggested by Morse [12] and Morse & Holzenthal [15]: absence of the primitive phallic parameres, reduction of the phallicata, and presence of a large tooth on each pupal mandible. The tibial spur formula ranges from 0,0,0 (in *Amazonatolica *Holzenthal & Oliviera Pes [29]) to 2,2,4. It was previously stated that the Australian species *Westriplectes albanus *(Mosely) has two pre-apical mid-tibial spurs, making the spur formula 2,4,4. This was pointed out as erroneous by Neboiss [30], but the information is still used as a character for separating Triplectidinae from Leptocerinae, *e.g*. in Morse & Holzenthal [15] and Yang & Morse [23].

#### Subfamily Leptocerinae

Proposed synapomorphies for Leptocerinae are absence of the third fork in the hind wings (as undivided M1+2), the absence of hind wing crossvein s, and the absence of mid-tibial pre-apical spurs [12]. The first two characters are also present in members of Grumichellini [29,31,32] and the latter character was discussed previously.

### Methods background

The taxonomy of the family has mainly been based on adult morphology, mostly of males, and the classification in use today is predominantly based on that of Morse [12]. The most commonly used characters for generic and species delimitation are the tibial spur formula, wing venation and genital morphology. No molecular analyses of the family have previously been undertaken, except one study within the Grumichellini [31]. The use of molecular data for recovering phylogenetic hypotheses is rapidly becoming a common practice in taxonomy and systematics, and for Trichoptera such data have been used on levels ranging from the entire order [20-22], to families [33-36], subfamilies [37], genera [31,38-40] and single species [41,42].

To examine the robustness of the traditional Leptoceridae classification, we present a first molecular-based phylogenentic hypothesis of the family. The analyses are based on sequences from five different genes; comprising the mitochondrial Cytochrome oxidase subunit 1 (COI) and the four nuclear markers Cadherin-like gene (CAD), Elongation factor-1α (Ef-1α), RNA polymerase II (POL) and Isocitrate dehydrogenase (IDH), with the latter successfully used for resolving phylogenies within Lepidoptera [43] but not for Trichoptera. In light of these results, we propose some changes to the current classification within the family, including the elevation of two tribes to subfamily status and the synonymisation of two genera with two other genera.

## Methods

### Taxon sampling

Out of the 47 described extant genera of Leptoceridae, we collected, extracted and sequenced representatives of 35 genera (Additional file 1), including an undescribed genus from Madagascar. The data comprise representatives from all recognised tribes as well as some genera of uncertain tribal placement (*e.g. Brachysetodes *Schmid and *Poecilopsyche *Schmid). In most cases, at least two species from each genus and in the case of monotypic genera two specimens from each species were included. Two widespread genera (*Oecetis *and *Triplectides*) were more extensively represented in the analysis to add as much genetic variation as possible. The outgroups were chosen based on previously published phylogenetic hypotheses, *e.g*. Kjer *et al. *[21] and Holzenthal *et al. *[3,22], and include all closely related and some more distantly related families. Vouchers are stored in alcohol at the Swedish Museum of Natural History (NHRS).

### DNA extraction & sequencing

Laboratory procedures follow those outlined in Johanson & Malm [35] for the fragments of the four genes: *COI, EF-1α, CAD *and *POL*. PCR reactions for the *IDH *fragments were performed with the primer pair deg27F-ino GGW GAY GAR ATG ACI AGR ATH ATH TGG and degR-ino TTY TTR CAI GCC CAI ACR AAI CCI CC, both modified after Wahlberg & Wheat [43], using the same protocol as the other genes, except with an annealing temperature of 52°C. Sequencing was accomplished using the same primer pairs as in PCR.

### Sequence analyses

The sequences of the *IDH *gene included a 15 bp length variable region, due to insertions/deletions of amino acids (basepair triplets), and were aligned using the L-INS-i method in the software MAFFT [44]. The sequences from each of the other genes were length invariable with disregard to missing bases in the beginning and in the end. These fragments were easily aligned using ClustalW [45] in the BioEdit Sequence Alignment Editor [46].

The sequence data were analysed using both maximum parsimony (MP) and Bayesian inference (BI). Analyses were performed of the separate genes as well as of combined datasets, analysed with and without exclusion of genes and of taxa lacking sequence data for more than one gene fragment (*i.e*. voucher DB2 and DO5) (datasets are available on request from TM).

The MP analyses were performed in TNT 1.1 [47] using 4,000 replicates of the 'xmult' search coupled with one 'drift' and one 'ratchet' search, followed by an additional 'ratchet' search and a final branch swapping. Multiple trees were collapsed to a strict consensus tree by the nelsen algorithm. To measure MP support a Jackknife analysis of each data set was performed with 4,000 replicates, each with 10 pseudo-replicates.

The BI analyses were carried out using MrBayes 3.1 [48] on Bioportal http://www.bioportal.uio.no. All analyses were run for 60,000,000 generations and sampled every 3,000 generations. The first 5,000 (out of 20,000) trees of each analysis were discarded as burn-in after visual inspection in Tracer v4.1 [49]. The Bayesian analyses of the single genes as well as the concatenated data set were performed in two separate ways: one with each gene fragment treated as one partition and one with each codon position of each gene sequence treated as one partition. For each of these partitions, the best nucleotide substitution models were determined using MrModeltest 2.2 [50] under the AKAIKE criterion. The models thus invoked in the analyses were for all partitions GTR + I + G, except *EF-1α *codon position 2 (F81 + I + G) and *POL *codon position 2 (HKY + I). The parameters of character state frequencies, substitution rates, proportion of invariable sites, and gamma shape were unlinked across the different partitions for all analyses.

For discussions about clade support, we regarded clades supported by both MP and BI analyses as more strongly corroborated than those supported by only one analysis method.

For conservative interpretation of the molecular phylogeny, equally large samples of trees (exclusive of burnin) were extracted from the posterior distributions of analyses run under different parameter settings (*i.e*. previously explained partition settings) and subjected to majority rule consensus calculation. This composite posterior distribution of trees allows for the most commonly existing groups from the variety of posterior distributions to be presented in the final phylogeny, irrespective of presence in one or more single analysis trees. This may result in less resolution than the single analysis posterior phylogenies, but the resulting groups could be interpreted as more stable.

### Graphical display

Trees were graphically enhanced in the software TreeGraph2 v. 2.0.40-184 beta http://treegraph.bioinfweb.info/ and figures were drawn and put together in the program InkScape v. 0.46 http://www.inkscape.org.

### Morphology

Terminology of body and wings structures follow that of Holzenthal *et al. *[3] and of genitalia that of Morse & Neboiss [51].

### Deposition of hard copies

To follow the regulations of the International Code of Zoological Nomenclature we have deposited copies of this article at the following publicly accessible libraries: Bombay Natural History Society, Mumbai, India; Koninklijk Belgisch Instituut voor Natuurwetenschappen, Brussels, Belgium; the Natural History Museum, London, UK; American Museum of Natural History, New York, USA; Museum national d'Histoire naturelle, Paris, France; Russian Academy of Sciences, Moscow, Russia.

## Results

### Single genes

The analyses using BI of the single gene datasets (not shown), with one or three partitions, were all largely displaying the same basal groupings within Leptoceridae, recovered as monophyletic in all but the *IDH *and *POL *genes. Both genes placed *Leptorussa *Mosely outside of Leptoceridae. The four monophyletic clades (1) *Leptorussa*, (2) Grumichellini, (3) Leptocerinae (excluding *Leptorussa*) and (4) Triplectidinae (excluding Grumichellini) were consistently recovered, except in the *POL *analyses where *Poecilopsyche *was recovered within Triplectidinae.

The monophyly of Grumichellini was recovered as (*Grumichella *Müller,(*Atanatolica*,(*Triplexa, Gracilipsodes *Sykora))) in the separate *EF-1α *and *IDH *analyses, as (*Atanatolica*,(*Grumichella*,(*Triplexa, Gracilipsodes*))) in the *CAD *and *POL *analyses, and for *COI *as ((*Grumichella, Atanatolica*),(*Triplexa, Gracilipsodes*)).

In all analyses, except those of the *COI *sequences, the subfamily Triplectidinae (excluding Grumichellini) was divided into Hudsonemini and Triplectidini. In the analysis based on the *COI *sequences the subfamily was monophyletic (with exclusion of Grumichellini), but with an internal basal collapse. *Condocerus paludosus *Neboiss was consistently found as sistergroup to *Hudsonema flaminii *Navás, within *Hudsonema*, which in the separate analyses of *IDH*, *COI *and *CAD *also included *Notalina. Symphitoneuria *and *Triplectidina *were recovered as polyphyletic in all single gene analyses and *Triplectides *was recovered as polyphyletic in the *COI *analysis.

Monophyly of Leptocerinae (excluding *Leptorussa*) was supported in all separate analyses, but with no major congruencies in internal topology among them. Some clades within Leptocerinae were however more stable, *e.g*. a monophyletic Oecetini with *Oecetis *including *Ptochoecetis *Ulmer; a monophyletic Triaenodini; a group consisting of *Setodes flagellatus *Gibbs basally of Mystacidini + Setodini; Athripsodini *s.str*. with *Ceraclea *and *Athripsodes *+ *Homilia lardeuxi *Gibon. The new Madagascaran genus was found as sister taxon to *Blyzophilus *in the *COI *and *CAD *analyses. *Poecilopsyche *forms the sister group to *Brachysetodes *in the *COI *analyses, to *Athripsodes *in the *EF-1α *analyses and to *Notalina *in the *POL *analyses. All genera except those mentioned above were recovered as monophyletic in all single gene analyses.

The MP analyses of the separate genes (not shown) produced trees generally congruent with the trees from the BI analyses, finding the same four basal groups, but generally the consensus trees were less resolved. *Poecilopsyche *was recovered within Leptocerinae in the *POL *analysis, in contrast to the placement in the BI *POL *analyses.

### Combined data

**Figure 3:**
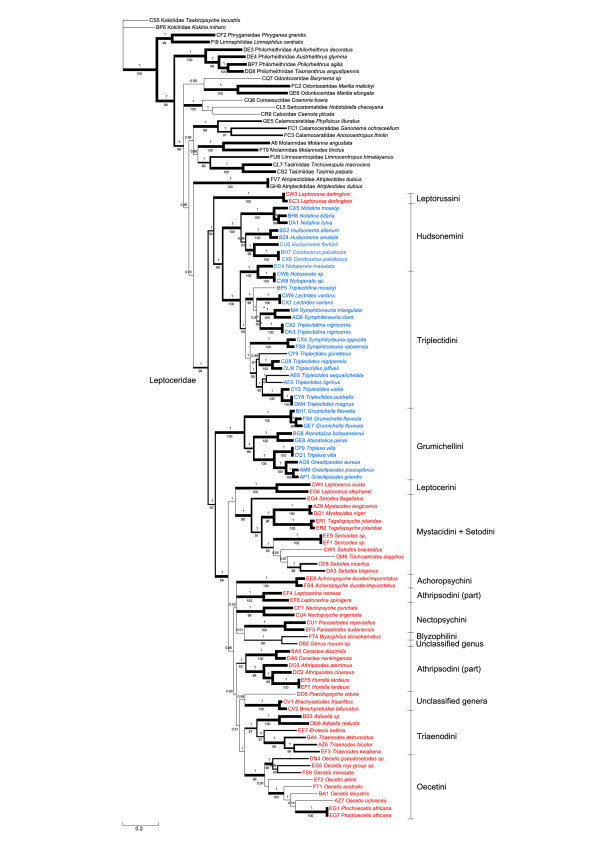
**Bayesian Inference (BI) topology of the complete data set, composite of two differently partitioned analyses**. BI composite phylogram from two separate analyses of the complete data set (including all taxa and genes), gene and codon position partitioned, respectively. Posterior tree samples merged for majority rule consensus tree. Values above branches correspond to clade frequency values (posterior probabilities, PP) in the composite tree posterior sample, and below branches to MP jackknife support indices. Branch lengths follow the BI analyses. Branch thickness corresponds to support, in four classes, thickest (PP = 1 and jackknife>95%), next thickest (PP>0.98 and jackknife>85%), next thinnest (PP>0.50 and jackknife>50%) and thinnest (only BI support). Colours of taxon names follow the presently used classification, Leptocerinae = red and Triplectidinae = blue.

The phylogenies derived from the BI analyses of the gene-partitioned data set (5 partitions) and the gene-codon partitioned data set (15 partitions), did not differ except in arrangement within the Leptocerinae, as indicated by the weak posterior probabilities in the composite majority rule consensus tree in Figure 3. The Leptoceridae was recovered as monophyletic and supported by both MP and BI analyses. Leptoceridae diverges into two clades: (1) *Leptorussa *+ Triplectidinae (excluding Grumichellini) and (2) Grumichellini + Leptocerinae (excluding *Leptorussa*). The former clade is weakly supported by MP but with high posterior probability values, presumably reflected by the differences in the placements of *Leptorussa *among the genes, but the latter is better supported by both methods. The subfamily Triplectidinae (excluding Grumichellini) consists of the two monophyletic tribes Hudsonemini and Triplectidini. Within the tribe Hudsonemini, *Notalina *forms a sistergroup to the well supported *Hudsonema*, including *Condocerus paludosus*. The Triplectidini is divided into a group containing *Triplectidina *(polyphyletic), *Lectrides *and two New Caledonian *Symphitoneuria *species; and one group consisting of two Tasmanian and Indonesian *Symphitoneuria *species as sistergroup to a monophyletic *Triplectides*. The tribe Grumichellini is, with support from both analysis methods, recovered as sistergroup to the subfamily Leptocerinae (excluding *Leptorussa*) and shows a well supported branching pattern of *(Grumichella, (Atanatolica, (Triplexa, Gracilipsodes)*.

The monophyly of Leptocerinae (excluding *Leptorussa*) (Figure 3) is well supported, but with many internal clades weakly supported. The clade (*Leptocerus*, (*Setodes flagellatus*, (Mystacidini, Setodini))) forms the sistergroup to a weakly supported group containing the remaining Leptocerinae. The monophyly of *Leptocerus *is strongly supported and of Mystacidini + Setodini well supported. Monophyly of *Setodes *is not supported as it includes *Trichosetodes *as well as excludes *S. flagellatus*. Among the remaining Leptocerinae the new genus groups together with *Blyzophilus*, though not very well supported. These two form a weakly supported clade together with *Parasetodes *McLachlan, *Achoropsyche*, *Nectopsyche *Müller and *Leptocerina*. The clade comprising Athripsodini *s.str*.: *Ceraclea *+ *Athripsodes *(including *Homilia lardeuxi*), is weakly supported as sistergroup to the remaining Leptocerinae. The weakly supported *Poecilopsyche *and *Brachysetodes *clade splits from a clade containing the two well-supported tribes *Triaenodini*, including (*Adicella *McLachlan, (*Erotesis, Triaenodes*)), and *Oecetini*, with *Oecetis *including *Ptochoecetis*.

**Figure 4:**
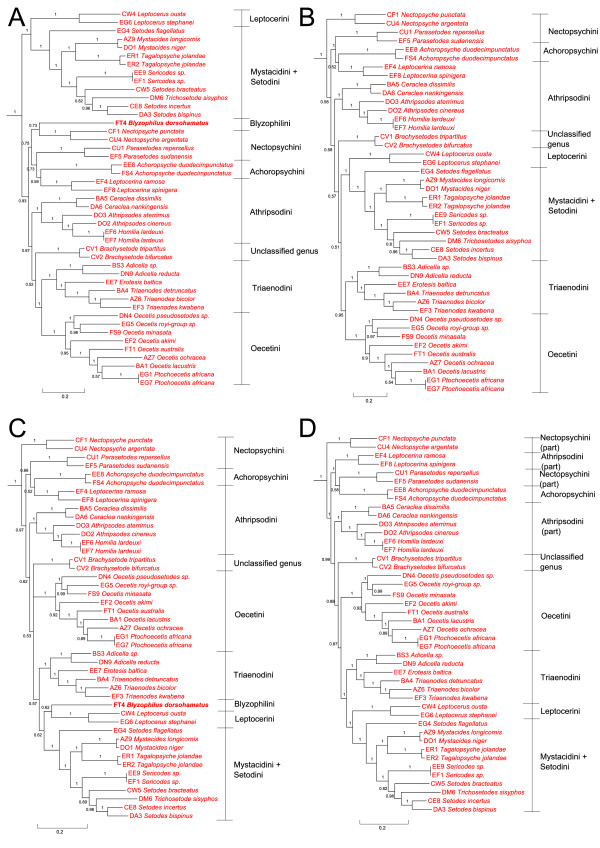
**Topology variation within subfamily Leptocerinae with exclusions of taxa and genes**. BI composite phylograms (from gene and codon position partitioned data) showing the topology variation inside Leptocerinae among analyses with different taxon and gene sampling. Taxa outside of Leptocerinae were cut off prior to presentation. Colours of taxon names follow Figure 3. **Figure 4A**. BI phylogram of a combined data set without the two taxa corresponding to NHRS vouchers DB2 and DO5. Support values above branches correspond to posterior probability values (PP). **Figure 4B**. BI phylogram of a combined data set without the three taxa corresponding to NHRS vouchers DB2, DO5 and FT4. Support values above branches correspond to posterior probability values (PP). **Figure 4C**. BI phylogram of a combined data set without the *COI *sequences and the two taxa corresponding to NHRS vouchers DB2 and DO5. Support values above branches correspond to posterior probability values (PP). **Figure 4D**. BI phylogram of a combined data set without the *COI *sequences and the three taxa corresponding to NHRS vouchers DB2, DO5 and FT4. Support values above branches correspond to posterior probability values (PP).

Analyses using BI of data sets with and without the exclusion of taxa missing more than one sequence (DB2 *Genus novum *and DO5 *Poecilopsyche vidura *Schmid) coupled with the exclusion/inclusion of the mitochondrial *COI*, yielded phylogenies consistent with previously described analyses within Triplectidinae, Grumichellini and Leptorussini, but differing greatly topologically within the subfamily Leptocerinae (Figure 4). *Blyzophilus *is either found in the clade consisting of *Nectopsyche*, *Parasetodes *and *Achoropsyche *(Figure 4A) or as sister to *Leptocerus *(Figure 4C). With the *COI *sequences excluded, the Leptocerini-Mystacidini-Setodini clade is recovered next to Triaenodini. Subsequent analyses without *Blyzophilus*, to eliminate the most topologically unstable taxon, place the Leptocerini-Mystacidini-Setodini clade next to Triaenodini (Figure 4D), or as sister to Triaenodini + Oecetini (Figure 4C).

**Figure 5:**
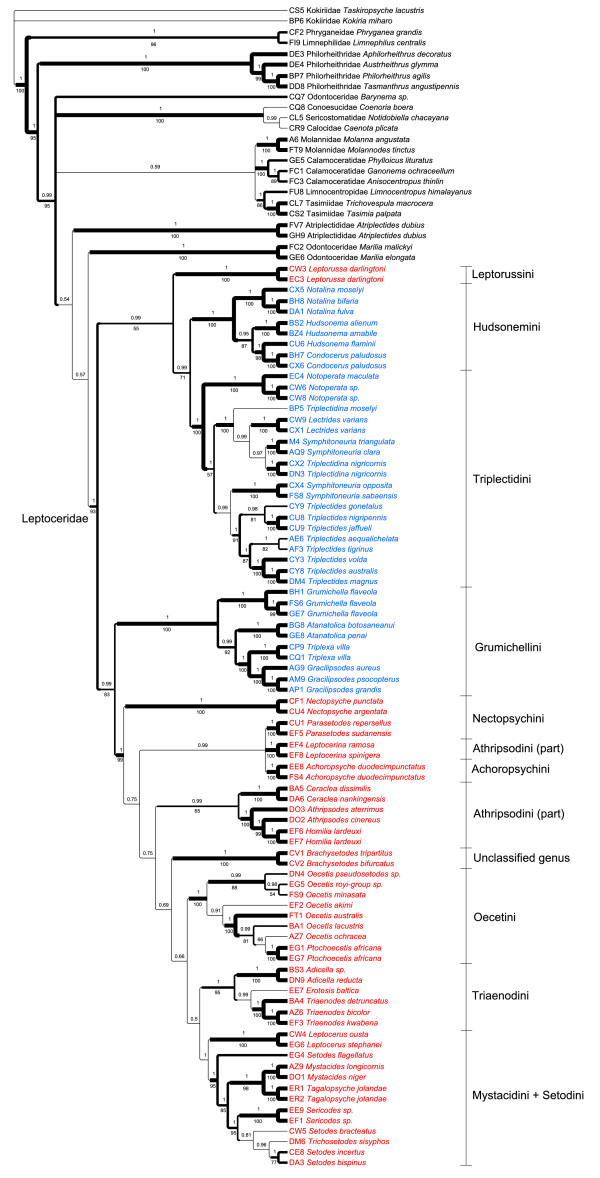
**BI composite topology of 4 different partitioned analyses**. BI composite topology of a data set *a priori *excluding 3 taxa (NHRS vouchers DB2, DO5 and FT4), partitioned in 4 different ways - gene and codon position partitioned and with or without the *COI *sequences. Values above branches correspond to clade frequency values (posterior probabilities, PP) in the composite tree posterior sample, and below branches to MP jackknife support indices. Branch lengths follow the BI analyses. Branch thickness corresponds to support, in four classes, thickest (PP = 1 and jackknife>95%), next thickest (PP>0.98 and jackknife>85%), next thinnest (PP>0.50 and jackknife>50%) and thinnest (only BI support). Colours of taxon names follow Figure 3.

The combination of 30,000 trees from each BI analysis of four different combined nuclear datasets (gene and codon partitioning, with and without mitochondrial *COI*), in a composite majority rule consensus of 120,000 trees (Figure 5), yielded a highly similar phylogeny to that of all taxa and all genes (see Figure 3), except within Leptocerinae. For this composite consensus analysis, three taxa were excluded a priori: DB2 *Genus novum *and DO5 *Poecilopsyche vidura*, both lacking sequence data for more than one gene fragment, as well as FT4 *Blyzophilus dorsohamatus *Andersen & Kjaerandsen, which proved to be the most topologically unstable taxon in previous analyses. In this analysis, within Leptocerinae, *Nectopsyche *is recovered as sistergroup to all other leptocerines, followed by a clade comprising *Parasetodes*, *Leptocerina *and *Achoropsyche*. Athripsodini *s.str*. is the sistergroup to the remaining leptocerines, where *Brachysetodes *is recovered as sister clade to (Oecetini, (Triaenodini, (Leptocerini, (*Setodes flagellatus*, (Mystacidini, Setodini))))). None of the basal branching events within Leptocerinae are strongly supported, as in the previous analyses.

The MP analysis (not shown) of the full combined data sets shows highly similar topologies as that given by the BI analyses, but with minor differences within Leptocerinae.

## Discussion

**Figure 6:**
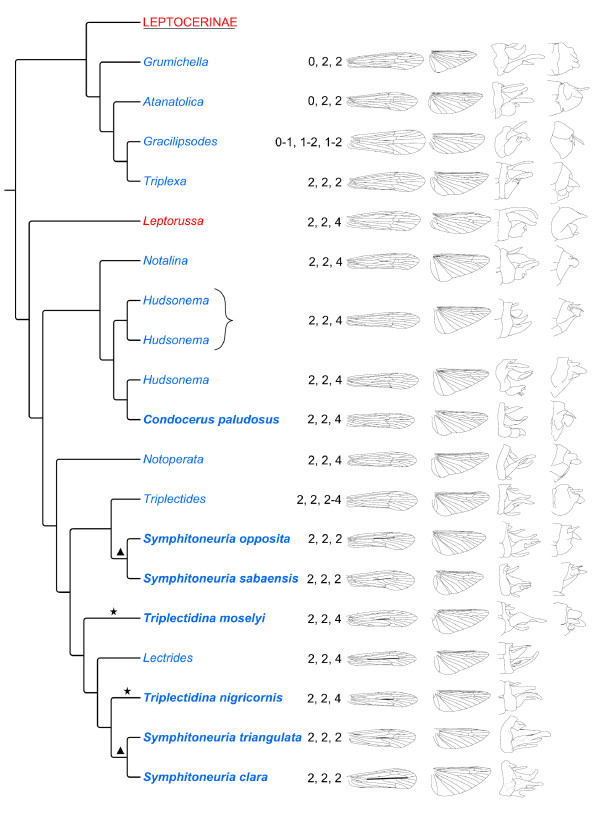
**New phylogeny of the Grumichellini, Leptorussini and Triplectidinae, presented with morphological characteristics**. Phylogeny based on the composite BI tree in Figure 5. The morphological characters tibial spur formula, forewing, hind wing, male and female genitalia, respectively, are presented next to the individual species or genera. Species names highlighted in bold are those contrasting with previous classifications (see text for detail). The triangle shows the placements of *Symphitoneuria *species and the star that of *Triplectidina *species. Colours of taxon names follow Figure 3.

**Figure 7:**
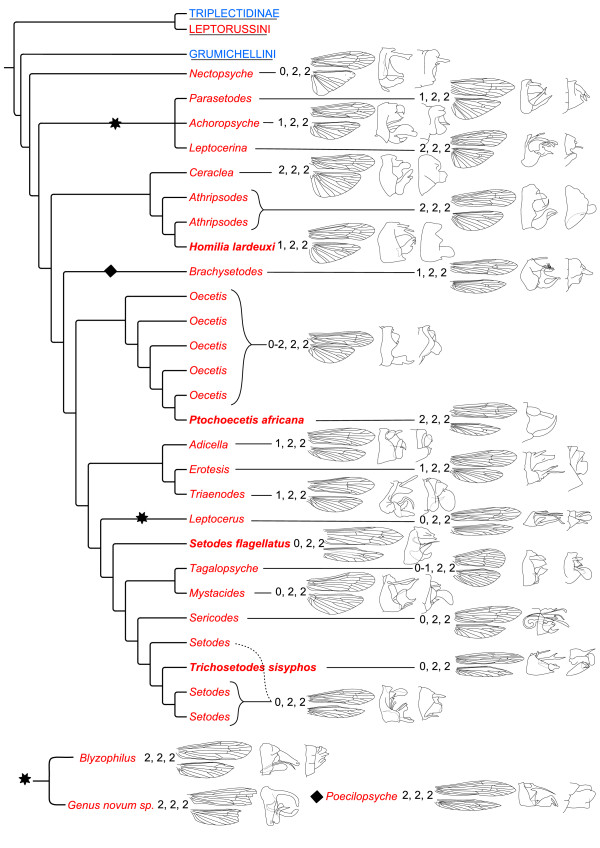
**New phylogeny of the Leptocerinae, presented with morphological characteristics**. Phylogeny based on the composite BI tree in Figure 5, with the addition of the genera *Blyzophilus *and *Genus novum *at two alternative placements corresponding to Figures 3 and 4. The morphological characters tibial spur formula, forewing, hind wing, male and female genitalia, respectively, are presented next to the individual species or genera. Species names highlighted in bold are those contrasting with previous classifications (see text for detail). The diamond shows the placement of *Poecilopsyche *according to Figure 3 and the star that of the two recovered positions of *Blyzophilus*+*Genus novum *according to Figures 3 and 4. Colours of taxon names follow Figure 3.

The results of these phylogenetic analyses (Figures 5, 6 and 7) corroborate earlier hypotheses of infra-Leptoceridae relationships based on morphological characters [6,12,15], but differ in some interesting and important aspects. The Leptoceridae apparently form a stable monophyletic group, even though a few single gene trees disagree (*i.e*. trees based on either *IDH *or *POL *BI and MP analyses). Even with the conservative approach used herein, of summarising data from more than one alternative run, we consistently recover the same four basal groups within the family: Leptorussini, Grumichellini, Triplectidini (excluding Grumicellini; hereafter Triplectidinae *s.str*.) and Leptocerinae (excluding *Leptorussa*; hereafter Leptocerinae *s.str*.).

*Leptorussa *(Leptorussini) forms the sister group to the Triplectidinae in all combined data trees. In some single gene trees, though, the position of the genus was placed outside Leptoceridae. Both these placements somewhat corroborates an earlier hypothesis of the genus as being very primitive within Leptoceridae [12]. This position is also corroborated by morphologiy, including the presence of the preapical hind-tibial spurs (Figure 6) and an apical fork 3 in the female forewing. The genitalia are unlike those of the Triplectidinae *s.tr *(Figure 6), *e.g*. with a spinelike phallus and very long superior appendages that are almost completely fused with tergum IX. The *Leptorussa *also lack the typical Triplectidinae characters of an enlarged anal region of the hind wings, as well as fork 3 in the male hind wing. Following these results, the tribe Leptorussini is elevated to form a monotypic subfamily Leptorussinae Morse, 1981.

The Grumichellini is recovered as monophyletic and distinct from the other Leptoceridae subfamilies. Usually it is recovered as sistergroup to Leptocerinae, however, in the *CAD *BI and MP gene trees it is found as the sistergroup to Triplectidinae *s.str*. + *Leptorussa*. The placement of Grumichellini as the sistergroup to the Leptocerinae is supported by shared morphological character states, *e.g*. a reduced tibial spur formula, 0,0,0 - 2,2,2, and the absence of the discoidal cell in the hind wings (except in *Triplexa*) (Figures 6 and 7). The genitalia resemble those of Triplectidinae, *e.g*. lacking phallic parameres (present in Leptocerinae), which may be a plesiomorphic trait (Figure 6). Holzenthal [26] listed 13 synapomorphies for Grumichellini larvae, of which many are not seen elsewhere in Leptoceridae. Grumichellini is elevated to subfamily Grumichellinae Morse, 1981, based on these results.

The Triplectidinae *s.str*. is consistently recovered as monophyletic, except for an aberrant placement of *Poecilopsyche *in the *POL *BI trees. The subfamily is divided into two tribes: Hudsonemini (*Hudsonema*, *Notalina *and *Condocerus*) and Triplectidini (*Notoperata *Neboiss, *Symphitoneuria*, *Triplectides*, *Triplectidina *and *Lectrides*). The species in Hudsonemini all lack harpagones and the basoventral lobe on the inferior appendages in the genitalia (Figure 6), and have unmodified wings (Figure 6) compared to in Triplectidini, which is characterised by having harpagones and basoventral lobes in the genitalia (Figure 6), and with modified R-M-Cu of the male forewings (but unmodified in *Notoperata*) (Figure 6). *Condocerus paludosus *(the type species of the genus) is always found in close conjunction with *Hudsonema flaminii *within *Hudsonema*. The only major difference between the two genera is the absence or presence of the crossvein s in the hind wings (Figure 6), and *Condocerus *is therefore considered being a junior synonym of *Hudsonema*, relative to these results. Both *Symphitoneuria *and *Triplectidina *are recovered as polyphyletic and in need of further taxonomic attention. The placement of the Tasmanian and Indonesian *Symphitoneuria *(*S. opposita *Walker and *S. sabaensis *Andersen & Huisman) next to *Triplectides *is interesting with regard to the wing venation within the tribe, as *Symphitoneuria*, *Lectrides *and *Triplectidina *all share similar strong modifications of the male forewing venation, which is much less pronounced in Triplectides (Figure 6). *Triplectidina *should be merged with *Lectrides *and the New Caledonian *Symphitoneuria *for easiest emendation, but more species of the group, with representatives from *Westriplectes *Neboiss and *Symphitoneurina*, are needed before final conclusions can be made. Thought of as being non-monophyletic by Morse & Holzenthal [15], *Triplectides *forms a monophyletic clade in all analyses, except in some of the single gene trees (*i.e*. the *COI *BI and MP analyses and the codon position partitioned *CAD *BI analysis).

Leptocerinae *s.str*. is recovered as monophyletic, except for the aberrant placement of *Poecilopsyche *in the *POL *BI analyses, but usually with weak internal resolution. The various analyses and datasets give entirely different hypotheses of the major branching pattern within the subfamily. Some groups are, however, consistently recovered in this subfamily: the group consisting of *Ceraclea*, *Athripsodes *and *Homilia *(hereafter Athripsodini *s.str*.); Oecetini consisting of *Oecetis *and *Ptochoecetis*; Triaenodini consisting of *Triaenodes*, *Adicella *and *Erotesis*; and the group with Setodini (*Setodes*, *Sericodes *and *Trichosetodes*) and Mystacidini (*Mystacides *and *Tagalopsyche *Banks). All these groups appear stable and their monophylies are supported by morphological character states.

The most robust phylogeny of this subfamily is given in Figures 5, 6 and 7, for which three taxa were excluded before analysis due to lack of sequence data (*i.e*. DB2 *Genus novum sp. *and DO5 *Poecilopsyche*) or instability in placement between different analyses (*i.e*. FT4 *Blyzophilus*), both possibly inducing erroneous phylogenies. In this phylogeny *Nectopsyche *is found as the sistergroup to all other taxa in the subfamily, but without close relation to *Parasetodes*; the other genus included in tribe Nectopsychini, based on the reduction of the stems of the radial sector and M in the hind wings. *Parasetodes *seems more affiliated with *Achoropsyche *(alone in the Achoropsychini) and *Leptocerina *(member of the Athripsodini), but the position of *Nectopsyche *among these taxa in some analyses may indicate a closer relationship between all those four genera. Some of the analyses including *Blyzophilus *and the new genus place those two genera close together with this group as well (Figures 3 and 4). Athripsodini is not a monophyletic group according to these analyses, and should only be used as Athripsodini *s.str*. (*Ceraclea*, *Athripsodes *and *Homilia*). *Homilia lardeuxi *is found inside of *Athripsodes*, leading to the conclusion that this species (and perhaps other *Homilia *species) belong there. The generic difference between *Homilia *and *Athripsodes *has been the absence of one of the foretibial spurs (1,2,2 from 2,2,2) (Figure 7), a trait that may have evolved within *Athripsodes*, but more species of both genera are needed to clarify the status of *Homilia. Brachysetodes *and *Poecilopsyche *are, as hypothesised by Morse [12] (Figure 1), not closely related to any particular tribe but form a clade basal to the "higher Leptocerinae", sharing the feature of having narrow hind wings (Figure 7). In our phylogenetic hypothesis, the group of "higher Leptocerinae" corresponds well with Morse's [12] opinion (Figures 1 and 7), except in the placement of the two tribes Leptocerini and Oecetini. The Oecetini clade correctly includes *Oecetis *and *Ptochoecetis*, differentiated by the absence of a fork on the M vein in the hind wing of the latter (Figure 7). *Ptochoecetis africana *(type species of the genus) is recovered deeply inside *Oecetis*, leading to the conclusion that *Ptochoecetis *is a junior synonym of *Oecetis. Ptochoecetis africana *is highly similar to certain *Oecetis *species in the genitalia (*e.g. O. bicuspida*) (Figure 7). The tribe Oecetini is recovered as sistergroup to the (Triaenodini, (Leptocerini,(*Setodes flagellatus*,(Mystacidini, Setodini)))) clade, which species share the character state of a posteriorly extended sternum IX (Figure 7). In our phylogeny, Triaenodini follows the current classification with *Adicella *as the sister genus to *Erotesis *and *Triaenodes*. The tribe Leptocerini is recovered as sister to Setodini and Mystacidini, but in some of the analyses (Figure 4C) Leptocerini is more closely related to *Blyzophilus *than to Setodini and Mystacidini, as hypothesised by Andersen *et.al. *[16]. The tribe Mystacidini, including *Tagalopsyche *and *Mystacides*, is strongly supported as being monophyletic and forming the sistergroup to Setodini. A number of species of those two genera share with the Setodini the reduction of the stem of the radial sector (stem of R2-5) in the hind wings (Figure 7). The interesting placement of *Setodes flagellatus*, a very non-'*Setodes*-like' species, and the inclusion of *Trichosetodes sisyphos *Malicky & Prommi within *Setodes*, indicate some uncertainty in the status of these genera. By these results, a new genus should be erected for *Setodes flagellatus*, and *Trichosetodes *should be synonymised with *Setodes*, the latter solution already indicated by Schmid [17]. But, in order to conclude anything about the stability and phylogenetic position of these taxa with certainty, more species of the genera (as well as type species) and of the affiliated genus *Hemileptocerus *must be included in the analysis.

Regarding the morphological characters most commonly used for identification and systematic placement of species within Leptoceridae, *i.e*. tibial spur formula, wing venation and male genitalia, Figures 6 and 7 show that these characters systems are overall consistent within the various tribes and also between tribes, but that apomorphic changes often occur. The number of tibial spurs characterises the basal split within Leptoceridae, with the Leptorussini + Triplectidinae having a pair of pre-apical hind-tibial spurs (reduced in some higher triplectidines) that are lacking in Grumichellini and Leptocerinae. The spur numbers are also fairly consistent within subfamilies and tribes, but often with reductions in number among the higher clades. Thus, not too much weight should be given to singular reductions in spur numbers as generic characters, as they can evolve within generic lineages, *i.e*. shown for *Gracilipsodes *by Malm & Johanson [31] and as may be the case with *Homilia *within *Athripsodes*. For wing characters, according to these findings, is the lack of crossvein s in the hind wing not a synapomorphy for Leptocerinae as earlier proposed, but a common character state within the family. The crossvein is present only in Triplectidinae and *Triplexa *(Grumichellini). Wing characters are often highly informative on family and genus level, but singular modifications in wing venation such as reductions of apical forks and reductions in spur number may not be enough to warrant generic status as they may be apomorphic adaptations within an existing genus (*i.e. Ptochoecetis*). Many genitalic characters are hard to track through the entire tree and are usually most applicable on tribus to species level, but some characters may be reliable at deeper levels, *i.e*. the harpagones within Triplectidinae for separating Hudsonemini from Triplectidini.

## Conclusions

This work strongly supports the monophyly of most of the previously classified groups within the family based on morphology, and most of the morphological characters discussed herein correspond well with our molecular-based phylogenies. But as stated previously, too much weight should not be laid on singular spurious modifications - whether in wing or body characters - when describing new higher taxa.

Our molecular-based phylogenetic hypotheses are robust at the subfamily level as well as the tribal level within Triplectidinae, but some questions remain unresolved. The utility of yet another gene for resolving the infra-familiar relationships of Leptocerinae would probably give less impact than better taxon sampling, since most of the genes show the same intra-relationships. The inclusion of *Nietnerella *in future analyses will answer the question whether or not to include that genus in the family. The importance of including specimens of the genera *Symphitoneurina *and *Westriplectes *in order to make final conclusions about the relationships within Triplectidini cannot be understated. Representatives of *Osflintia *and *Amazonatolica *may resolve the phylogeny of the Grumichellini. Most important, though, would a better taxon sampling within Leptocerinae allow more robust hypotheses about tribal and generic relationships to be made. Great advances would probably be made by including specimens of the missing genera *Amphoropsyche *Holzenthal, *Axiocerina *Ross, *Hemileptocerus, Leptecho *Barnard, *Leptoceriella *Schmid, *Neoathripsodes *Holzenthal and *Russobex *StClair. But even so, this work improves our understanding of the phylogeny and evolution within the Leptoceridae and its subgroups, and builds a new platform for further studies of the family.

### Proposed classification changes within Leptoceridae

**Grumichellinae **Morse, 1981, **status novum **- by removal of tribe Grumichellini from Triplectidinae.

**Leptorussinae **Morse, 1981, **status novum **- by removal of *Leptorussa *from Leptocerinae.

***Hudsonema ***Mosely, 1936 (= *Condocerus *Neboiss, 1977), **new synonym.**

***Hudsonema paludosa ***(Neboiss, 1977), **new combination **- necessitated by synonymisation of *Condocerus *with *Hudsonema*.

***Hudsonema apta ***(Neboiss, 1982), **new combination **- necessitated by synonymisation of *Condocerus *with *Hudsonema*.

***Oecetis ***McLachlan, 1877 (= *Ptochoecetis *Ulmer, 1931), **new synonym**.

***Oecetis corbeti ***Malm & Johanson, **nomen novum **- necessitated by synonymisation of *Ptochoecetis *with *Oecetis *and a resulting homonymy: *Oecetis africana *(Kimmins, 1957), a secondary junior homonym to *Oecetis africana *Ulmer, 1931; since the name *O. kimminsi *is preoccupied (Kumanski, 1979), the species is re-named for the collector of the holotype, Dr. Philip S. Corbet.

***Oecetis tenella ***(Navás, 1931), **new combination **- necessitated by synonymisation of *Ptochoecetis *with *Oecetis*.

## Abbreviations

**HKY + I**: Hasegawa-Kishino-Yano; 85 substitution model with invariable sites; **GTR + I + G**: general time reversible substitution model with gamma distribution and invariable sites; **F81 + I + G**: Felsenstein, 81 substitution model with gamma distribution and invariable sites; **R1-5**: radial veins 1-5; **M1+2**: median vein 1+2 (anterior median vein); crossvein **s**: sectoral crossvein connecting R2+3 with R4+5; **M**: median vein; **Cu**: cubital vein; ***s.str.***: *sensu stricto *(*Latin*: in the narrow sense); ***s.l.***: *sensu lato *(*Latin*: in the broad sense).

## Authors' contributions

TM was responsible for the major part of the molecular sequencing work, as well as most analyses and the manuscript draft. KAJ critically reviewed the manuscript and played a major part in the acquisition of specimens. Both authors have read and approved of the final manuscript.

## Supplementary Material

Additional file 1 **Specimen information with Genbank/EMBL accession numbers**. List of specimens sequenced and analysed in this work. Species names sorted under family affinity, with NHRS voucher code, information of gender/life stage and sample locality as well as accession numbers to online sequence databases. Click here for file
